# Ultrasound-Guided Lumbar Plexus-Sciatic Nerve Blocks Versus Epidurals for Orthopaedic Surgeries: A Study to Compare the Competency of Novice Anaesthesiology Residents in a High-Volume Level 1 Trauma Centre

**DOI:** 10.7759/cureus.69539

**Published:** 2024-09-16

**Authors:** Tanvir Samra, Ashish Aditya, Paritosh Kumar Amar, Kajal Jain, Vikas Saini, Naveen Naik B

**Affiliations:** 1 Anaesthesia and Intensive Care, Postgraduate Institute of Medical Education and Research, Chandigarh, IND

**Keywords:** epidural analgesia, lumbar plexus block, postoperative analgesia, ropivacaine, sciatic nerve block

## Abstract

Introduction: The relative merits of peripheral nerve blocks (PNB) over central neuraxial anaesthesia and the advantages of the above two techniques over general anaesthesia for surgical interventions of the lower limb are well established. The competency of anaesthetic trainees in a high-volume level 1 trauma centre in administering dual ultrasound and nerve stimulator-guided lumbar plexus-sciatic nerve block (DUNLuPS) vs. epidural anaesthesia (EA) was compared by reporting the adequacy of anaesthesia with the two techniques, time taken for the performance of block, time of onset of sensory block (TOSB), and time of onset of motor block (TOMB).

Materials and methods: This prospective, randomized, study enrolled 92 patients aged 18-80 years with lower limb fractures admitted in trauma triage and scheduled for surgery. The patients were randomly allocated equally into the EA group and the DUNLuPS group. A total of 20 anaesthesia trainees in the third year of residency with clinical experience of more than 15 independent lumbar plexus-sciatic nerve blocks were included in the study. A volume of 20 ml of 0.5% ropivacaine was administered in the lumbar plexus (Shamrock technique) but the volume used for sciatic nerve (subgluteal approach) was varied so that the cumulative dose did not exceed 3 mg/kg. For each block, the onset of nerve blockade was assessed every five minutes, and the assessments continued for an additional 30 minutes after the nerve blocks were finished.

Results: Clinical characteristics and adequacy of anaesthesia were comparable, i.e., 95.65% and 93.47% success in the EA (n = 46) and DUNLuPS (n = 46) groups, respectively. Performance time was significantly more in the DUNLuPS group but followed by significantly less TOSB and TOMB. The time for the first analgesic request was 351.63 ± 148.70 minutes in the DUNLuPS group and 147.60 ± 52.65 minutes in the EA group (p < 0.0001).

Conclusion: Both EA and DUNLuPS provide effective and comparable intra-operative anaesthesia for orthopaedic lower limb surgeries (OLLS) when administered by residents with more than two years of experience (third year of residency) in ultrasound-guided regional nerve blocks in a high-volume level 1 trauma centre. Statistically significant differences in the block performance characteristics had no clinical advantage as it was compensated by the faster onset time in the DUNLuPS group. Post-operative pain management was better in the DUNLuPS group, so the practice and conduct of anaesthesia for trauma patients should focus on the establishment of “block rooms” and timely training of residents in the former so that the advantages can be extended to the patient population.

## Introduction

There is an urgent need to train residents in the art of anaesthetizing trauma patients, especially in high-volume level 1 trauma centres, as they form the major proportion of the workforce in operating theatres [1]. In a structured curriculum for trauma anaesthesiology, the aim is to train the residents in non-technical (affective and cognitive domain) and technical skills (psychomotor domain) [2].

It is mandatory for a graduate of an accredited anaesthesia residency program in the United States to have provided care for 40 patients administering peripheral nerve blocks (PNBs) [3]. In our centre, around 500 trauma cases are being operated on in a month, and thus the trainee develops adequate cognitive and technical skills to administer both neuraxial anaesthesia (NA) and PNBs. The most cited advantage of PNB over epidural anaesthesia (EA) is the haemodynamic stability of the former [4]. Also, positioning for neuraxial blocks is challenging in post-trauma patients with external fixators in situ or with multiple fractures. Trauma-induced coagulopathy or dilutional coagulopathy following resuscitation precludes central neuraxial blockade in this subset [5].

The efficacy of lumbar plexus-sciatic nerve block over central neuraxial anaesthesia for lower limb orthopaedic surgery is well established [6,7]. Safety, reliability, predictability, ease of administration, and post-operative analgesia with the above two techniques, however, have not been compared when administered by the trainee in a high-volume trauma centre and this precludes the formulation of a consensus on the better choice among the two for orthopaedic lower limb surgeries (OLLS).

The primary aim of this study was to compare the success of anaesthesia of dual ultrasound and nerve stimulator-guided lumbar plexus-sciatic nerve block (DUNLuPS) versus EA when administered by adequately trained trainees in a high-volume trauma centre. The secondary aim was to compare the haemodynamic perturbations, first analgesic request time, degree of motor blockade, time of onset of sensory block (TOSB) and time of onset of motor block (TOMB), dynamic and static pain scores at six, 12, and 24 hours post-operatively, and opioid/non-opioid analgesic supplementation in first 24 hours after surgery.

## Materials and methods

This randomized controlled study was conducted in a level 1 trauma centre of a tertiary care institute from January 2019 to October 2019 after approval of the Institutional Ethics Committee (NK/4654/MD/563; dated: 21st September 2018). The trial was registered prospectively with the Clinical Trials Registry of India (CTRI/2019/01/017312). Patients aged 18-80 years with American Society of Anesthesiologists (ASA) physical status 1-3 and undergoing lower limb surgery were enrolled after written informed consent. Patients refusing regional anaesthesia, allergic to amide local anaesthetic drugs, on chronic opioid therapy, patients with an inability to understand the numeric rating scale (NRS), with a linguistic barrier, with thrombocytopenia/coagulopathy, on anti-coagulants and thrombolytic therapy, and patients with morbid obesity (BMI > 35 kg/m2 with obesity symptoms or BMI > 40 kg/m2), neurological diseases, infections in the intervention site, and psychiatric illness were excluded.

Randomization was done using a computer-generated randomization program (http://www.randomizer.org) and the patients were allocated into either the EA or DUNLuPS group using sequentially numbered sealed opaque envelopes. Trainee anaesthesiologists in the third year of residency posted in the advanced trauma centre with clinical experience of more than 15 independent lumbar plexus-sciatic nerve blocks and EA performed under the supervision of trauma anaesthesia faculty were selected for the intervention. EA or DUNLuPS was administered based on the group allocation of the patients. The anaesthesiologist performing the epidural injection/blocks was not involved in further intraoperative or post-operative management of the patient or collection of the data.

After ensuring fasting status, routine monitoring (electrocardiography, pulse oximetry, non-invasive blood pressure (NIBP)) was started in the operating room and a co-load of 500 ml of balanced salt solution was initiated for all the patients while starting the procedure. Supplemental oxygen was provided to all patients using nasal prongs at a flow rate of 4 litres per minute.

Patients were positioned laterally with the operative side up and appropriate aseptic precautions were taken. In the DUNLuPS group, ultrasound (US)-guided (Sonosite portable ultrasound unit, FUJIFILM Sonosite, Inc., Bothell, WA) lumbar plexus block using the Shamrock technique was administered. The plexus was identified as a hyperechoic round oval structure in the medial and posterior part of the psoas muscle and a 22-gauge, 150 mm nerve block needle (Stimuplex Ultra, B. Braun, Melsungen, Germany) was inserted towards the plexus. After eliciting an appropriate motor response to nerve stimulation with 0.3-0.5 mA from either the quadriceps femoris or the thigh adductors, 20 ml of 0.5% ropivacaine was given. The block was then supplemented with a US-guided sciatic nerve block using the subgluteal approach to achieve complete anaesthesia of the lower limb. The volume of 0.5% ropivacaine administered in the sciatic nerve block was adjusted so that the maximum permissible cumulative dose of ropivacaine did not exceed 3 mg/kg. In the EA group, the trainee identified the L3-L4 intervertebral disc space and under all aseptic conditions, after local skin infiltration with lignocaine, epidural space was identified using loss of resistance technique with saline. The catheter was advanced approximately 5 cm in the cephalic direction. A test dose of 3 ml of 2% lignocaine containing epinephrine (freshly added) in a ratio of 1:200,000 was administered. A negative test dose ruled out the intrathecal or intravascular catheter, which was then followed by incremental doses of 15 ml of 0.5% ropivacaine.

Assessment of success of the block

The sensory block was evaluated at the interval of five minutes and continued for 30 minutes post-intervention. Sensory evaluation was done bilaterally in the EA group and on the side of the block in the DUNLuPS group using a 21-gauge blunt needle. Loss of pinprick sensation was tested in the following nerve territories: sole of the foot (sciatic), anterior thigh (femoral), lateral thigh (lateral cutaneous), and medial thigh (obturator). The L1, T12, and T10 dermatomal levels were also tested in both groups at five-minute intervals. Successful sensory block is defined as complete loss of sensation to pinprick in all nerve territories for DUNLuPS and T10 dermatome for EA.

Motor block was evaluated by testing knee extension (femoral nerve), thigh adduction (obturator nerve), dorsiflexion, and plantar flexion of the foot (common peroneal and tibial nerves). The motor block intensity was assessed using the modified Bromage score (MBS) and a successful motor block was defined as an MBS of 3 at the hip, knee, and foot.

The success of anaesthesia with DUNLuPS and EA was defined as no complaint of intraoperative pain after initial successful motor and sensory block.

The time of onset of sensory block (TOSB) was defined as the time from completion of DUNLuPS or EA to the occurrence of complete sensory loss in the related nerve territories or up to T10 dermatome, respectively.

The time of onset of motor block (TOMB) was defined as the time from completion of DUNLuPS or EA to the time when MBS was 3 at the hip, knee, and foot.

Intra-operative sensory and motor block assessments were done every hour and when pinprick was positive at T12 dermatome or MBS < 3, an epidural bolus of 5 ml of 0.5% ropivacaine in the EA group was administered. Any patient in the DUNLuPS group complaining of pain was administered general anaesthesia and later excluded from the analysis.

Post-operative pain management

Pain intensity was assessed using the NRS, which is a 0-10 ranking scale with 0 representing “no pain” and 10 representing “unbearable pain”. Static and dynamic values of average, best, and worst NRS scores were recorded at admission to the post-anaesthesia care unit (PACU) (time 0) and at six, 12, and 24 hours. The time at which NRS was ≥3 was defined as the time to rescue analgesia.

Rescue analgesia

In the EA group, epidural analgesia was initiated with 7 ml of 0.2% ropivacaine and reassessed after 15 minutes. Failure of pain relief, i.e., NRS ≥ 3, after the first epidural bolus necessitated another bolus.

In the DUNLuPS group, all the patients were treated with intravenous diclofenac (75 mg, first-line rescue), except those suffering from chronic kidney disease (CKD) or allergic to diclofenac, where 1000 mg of paracetamol (PCM) was preferred.

Patients with persistent pain (NRS between 3 and 7) after diclofenac/PCM and two bolus doses of epidural analgesia in an hour were given tramadol 50 mg, and intravenous fentanyl 1 mcg/kg was administered if NRS ≥ 7.

Statistical analysis

Horasanli et al. reported a success rate of 92.5% with the use of peripheral nerve stimulation (PNS) for lumbar plexus-sciatic nerve block [6]. The success rate of epidural placement in our centre is 95%. We assumed that the use of US with PNS would increase the “success of providing adequate anaesthesia” in lumbar plexus-sciatic nerve block to that comparable for epidural, i.e., up to 95%. The sample size was calculated through a clinical sample size calculator. A total of 74 patients were required in both groups (37 each) at 80% power and 0.05 alpha level. Keeping in mind the dropout of samples in the course of the study, a sample of 46 was taken in each group.

Continuous quantitative variables are presented as mean ± SD or median and interquartile range; categorical or qualitative variables are represented as frequencies or percentages. The normality of the continuous data was checked using the Kolmogorov-Smirnov test. Parametric and non-parametric tests such as Student's t-tests, chi-square tests, and Mann-Whitney U tests were applied to look for significant differences. A p-value of less than 0.05 was considered significant.

## Results

**Figure 1:**
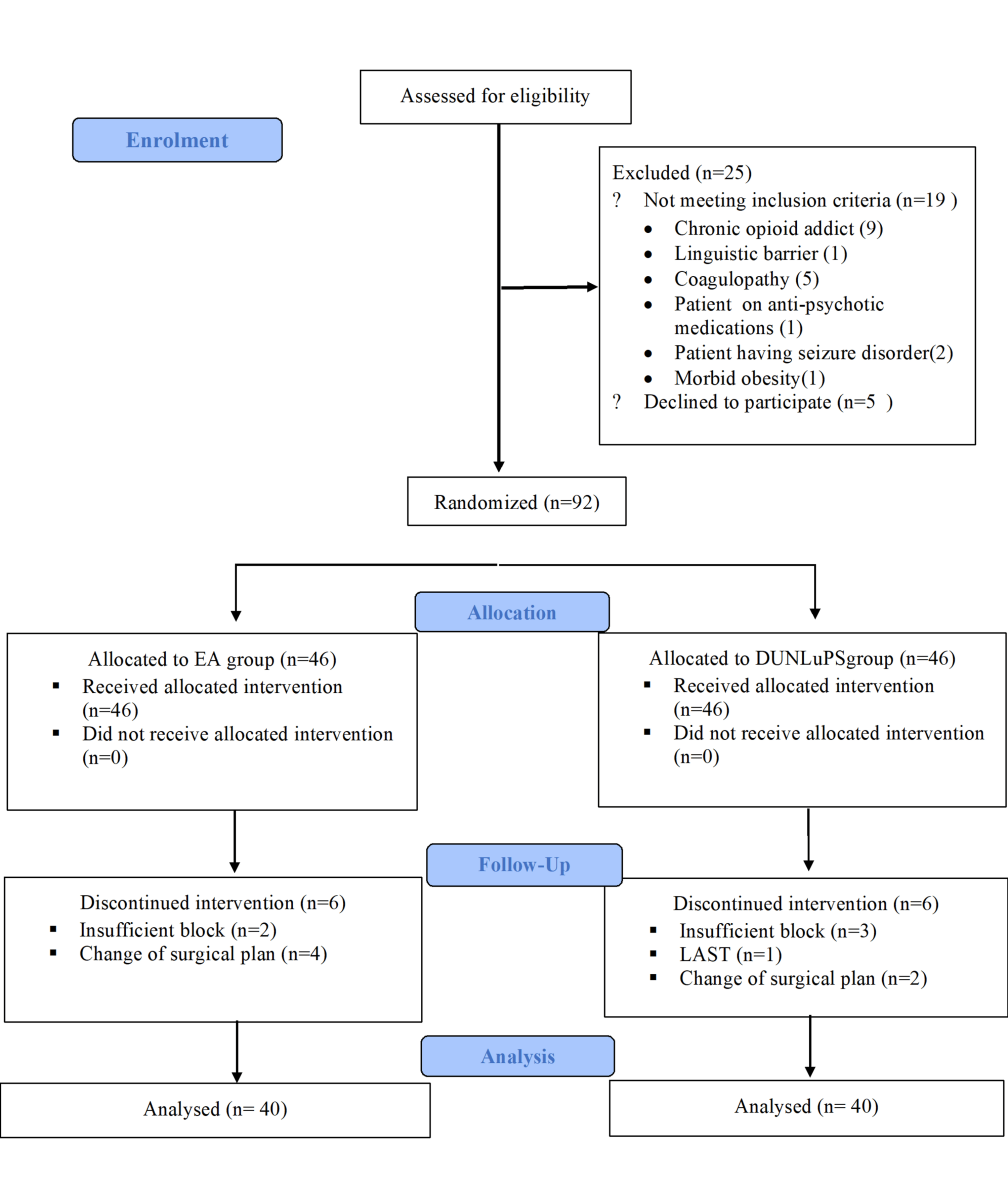
CONSORT flow diagram showing the participation of patients. CONSORT: Consolidated Standards of Reporting Trials; EA: epidural anaesthesia; DUNLuPS: dual ultrasound and nerve stimulator-guided lumbar plexus-sciatic nerve block.

A total of 117 patients were assessed for eligibility and 25 did not meet the inclusion criteria. A total of 92 patients were included in the study, with 46 allocated to each group. Six participants from each group were subsequently excluded, leading to the analysis of 40 patients in each group (Figure 1).

The mechanism of injury in all the participants was a roadside accident. Table 1 summarizes the demographic and clinical details of the patients. The majority of patients had undergone primary surgery for traumatic lower limb injuries, i.e., 72.5% in the EA group vs. 65% in the DUNLuPS group. Of the patients, 15% in the EA group and 17.5% in the DUNLuPS group were scheduled for second revision surgery, whereas 5% in the EA group and 7.5% in the DUNLuPS group were scheduled for third revision surgery. The majority of patients had both lower limb bone (tibia and fibula) fractures, i.e., 42.5% vs. 52.5% in the EA and DUNLuPS groups, respectively (Table 1).

**Table 1:** Demographic and clinical characteristics. ^! ^Four patients in the EA group were ASA 3, three patients had uncontrolled hypertension on medication, and one patient had uncontrolled DM and CKD (not on dialysis). ^@ ^Four patients in the DUNLuPS group were ASA 3, two had uncontrolled DM with CKD, and two had uncontrolled DM, CKD, and hypertension. None of the patients was on dialysis. * Values in mean (SD) and compared using Student's t-test. All other values are expressed as n (%) and all categorical variables are compared using the chi-square test. EA: epidural anaesthesia; DUNLuPS: dual ultrasound and nerve stimulator-guided lumbar plexus-sciatic nerve block; DM: diabetes mellitus; CKD: chronic kidney disease; HTN: hypertension; ASA PS: American Society of Anesthesiologists Physical Status; NYHA: New York Heart Association.

	EA group (n = 40)	DUNLuPS group (n = 40)	P-value
^*^Age (years)	37.53 (17.26)	34.00 (13.68)	0.32
Sex			0.64
Male	37	38	
Female	3	2	
^*^Weight (kg)	67.90 (7.12)	71.90 (8.58)	0.03
^*^Height (cm)	168.18 (8.52)	169.48 (6.56)	0.45
*BMI (kg/m^2^)	24.14 (2.88)	24.84 (2.01)	0.21
ASA PS			0.77
1	12 (30%)	15 (37.5%)	
2	24 (60%)	21 (52.5%)	
3	4 (10%)!	4 (10%)^@^	
NYHA class			0.72
1	31 (77.5%)	34 (85%)	
2	6 (15%)	6 (15%)	
3	2 (5%)^$^	0	
4	1 (2.5%)	0	
Co-morbid conditions			
HTN	7 (17.5%)	3 (7.5%)	0.18
DM	1 (2.5%)	4 (10%)	0.17
CKD	1 (2.5%)	4 (10%)	0.17
Anaemia	13 (32.5%)	13 (32.5%)	1.0
Smoker	7 (17.5%)	19 (47.5%)	0.004
Alcoholic	7 (17.5%)	12 (30%)	0.19
Surgical diagnosis			
Fracture of the tibia and fibula	17 (42.5%)	21 (52.5%)	
Fracture shaft of the femur	11 (27.5%)	2 (5%)	
Knee injury	6 (15%)	5 (12.5%)	
Fracture of the foot and ankle	6 (15%)	12 (30%)	
^*^Delay in admission to hospital (days)	1.65 (3.85)	3.48 (8.65)	0.228
*Delay in surgery (days)	6.98 (8.99)	9.30 (15.24)	0.409
*Duration of surgery (minutes)	233.65 (73.96)	215.88 (79.01)	0.302
*Duration of anaesthesia (minutes)	258.90 (75.17)	241.25 (81.62)	0.318
Number of previous surgeries	0.48 (0.91)	0.75 (1.39)	0.298

The total number of trainee anaesthesiologists fulfilling the criteria for inclusion in the study (third year of residency with clinical experience of more than 15 independent lumbar plexus-sciatic nerve blocks) was 20 in number. In the EA group, 95.65% of subjects achieved an MBS of 3 and a sensory block at T10. In the DUNLuPS group, 93.47% of subjects developed an MBS of 3 and sensory anaesthesia in the respective dermatomal and myotomal distribution. Thus, the failure rate was comparable in both groups: 4.34% and 6.52% in the EA and DUNLuPS groups, respectively (p = 0.34). The aggregate block performance characteristics of 46 epidurals performed in the EA group and 46 lumbar plexus followed by sciatic nerve blocks performed by the total 20 trainees rotated in the trauma centre are summarized in Table 2.

**Table 2:** Block performance characteristics. All values are shown in mean (SD) and compared using Student's t-test. * Values are shown in mean (SD). ^#^ TOSB and TOMB were considered for the EA group as the time after completion of administration of 15 ml of 0.5% ropivacaine in epidural space to onset of sensory block at T10 and MBS of 3. TOSB for the DUNLuPS group was considered after the completion of both blocks. It is to be noted that the lumbar plexus block was administered first and after that sciatic block was administered but TOSB was calculated after completion of both blocks. ^@^ Number of patients in the EA group who needed a single epidural top-up of 5 ml of 0.5% ropivacaine was 10 and four patients needed a second bolus of top-up for completion of surgery. EA: epidural anaesthesia; DUNLuPS: dual ultrasound and nerve stimulator-guided lumbar plexus-sciatic nerve block; MBS: modified Bromage score.

S. No.	Block performance characteristics	EA group	DUNLuPS group	P-value
1.	Time for performing procedure (minutes)	12.73 (3.64)	16.4 (4.55)	<0.001
2.	Total dose of ropivacaine administered intraoperatively (mg)^@^	85.37 (17.11)	185.25 (19.77)	<0.001
3.	Time of onset of sensory block (TOSB) in minutes​​​^#^	14.60 (5.13)	9.25 (3.50)	<0.001
4.	Time of onset of motor block (TOMB) in minutes​​​​​​^#^	20.60 (6.23)	14.38 (3.61)	<0.001

The average performance time of residents (labelled as A-T) is summarized in Table 3.

**Table 3:** Average block performance times. Trainees were labelled from A to T.

Trainee	Epidural anaesthesia performance time (minutes)	Trainee	Lumbar plexus block performance time (minutes)	Trainee	Sciatic nerve block performance time (minutes)
B	9	G, N, Q, R	5	I	5
E, I, P, R	10	J, K, D, M	6	D, Q	7
O, L	11	L	7	L, N, P	8
N, Q, G	12	C, H, I, P, S, F	8	G, K, R, T, M	9
F, T, S	13	T	9	B, C, E, H, O, S	10
H	14	B, E, O	10	F, J	11
A, J, M, D	15	A	14	A	13
K	19				
C	17				

**Figure 2:**
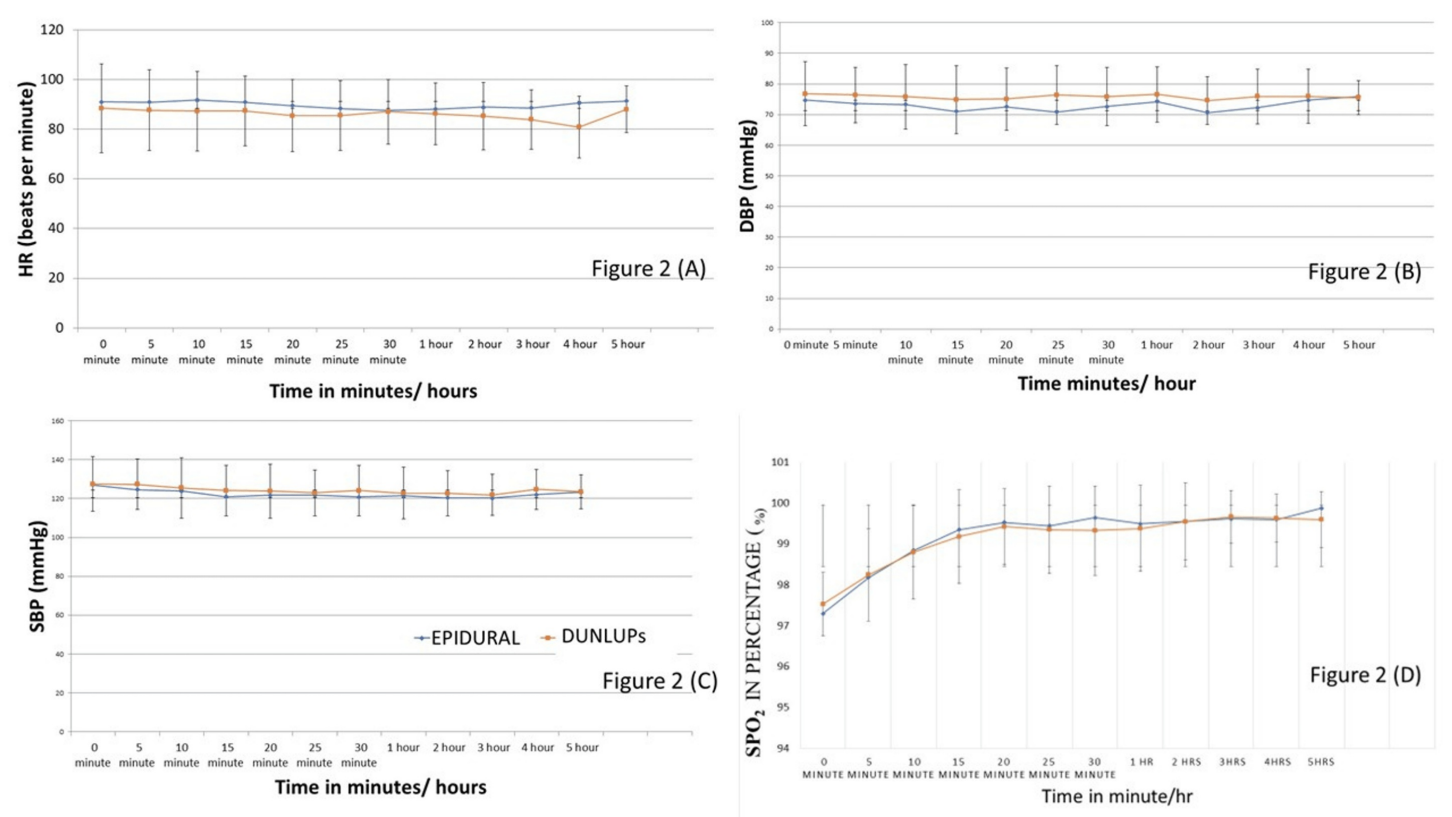
Intra-operative trends of vital parameters between the groups. A: Heart rate was comparable at all time points. B: DBP was significantly lower in the epidural group at a single time point of 25 minutes (p = 0.014). C: SBP was comparable at all time points. D: SPO2 was comparable at all time points. DUNLuPS: dual ultrasound and nerve stimulator-guided lumbar plexus-sciatic nerve block; HR: heart rate; DBP: diastolic blood pressure; SBP: systolic blood pressure; SPO2: oxygen saturation.

Intra-operative heart rate, blood pressure (BP), and oxygen saturation (SPO2) were comparable in both groups (Figure 2).

Eight out of 40 (20%) patients developed transient hypotension in the EA group, while only three patients (7.5%) developed transient hypotension in the DUNLuPS group; the difference was statistically not significant (p = 0.105, Pearson chi-square test). Transient hypotension was defined as systolic blood pressure (SBP) < 100 mmHg or a decrease of NIBP more than 20% from baseline in two consecutive readings of blood pressure at one-minute intervals. Hypotension was treated with 3 mg boluses of mephentermine or 1-1.5 mg/kg boluses of phenylephrine.

None of the patients in either group had a vascular injury, viscera perforation, dura puncture, bradycardia, infection, permanent neurological injury, retroperitoneal hematoma formation, or psoas abscess formation. Nausea and vomiting were reported in four (10%) patients in the EA group.

Post-operative data

Mean post-operative haemodynamic parameters were comparable. Motor block regression time was 186.98 ± 56.82 (mean ± SD) minutes in the DUNLuPS group and 93.33 ± 30.76 (mean ± SD) minutes in the EA group (p < 0.0001).

The time for the first analgesia request was 351.63 ± 148.70 (mean ± SD) minutes in the DUNLuPS group, which was significantly more (p < 0.0001) than that in the EA group, where it was 147.60 ± 52.65 (mean ± SD) minutes.

Post-operative pain scores are summarized in Table 4. In the EA group, 17 patients did not have adequate pain relief after bolus administration of local anaesthetic (LA) and were switched over to IV analgesics completely (10 were administered tramadol and seven were administered fentanyl) to maintain NRS ≤ 3.

**Table 4:** Static and dynamic pain score (NRS scores). * Values are shown in mean (SD) and compared using Student's t-test. Static or pain experienced at a single time point (e.g., pain threshold); and dynamic or changes in pain over time in response to sustained or repeated stimuli. EA: epidural anaesthesia; DUNLuPS: dual ultrasound and nerve stimulator-guided lumbar plexus-sciatic nerve block; NRS: numeric rating scale.

Time points	NRS score		EA group	DUNLuPS group	P-value
6 hours	Average	Static	2.375 (1.13)	1.9 (0.38)	0.013
		Dynamic	2.68 (1.33)	1.95 (0.39)	0.001
	Best	Static	1.6 (0.78)	1.075 (0.27)	<0.0001
		Dynamic	1.98 (1.05)	1.3 (0.46)	<0.0001
	Worst	Static	3.45 (1.45)	2.15 (0.43)	<0.0001
		Dynamic	3.83 (1.81)	2.3 (0.65)	<0.0001
12 hours	Average	Static	2.68 (0.99)	1.98 (0.36)	<0.0001
		Dynamic	3.1 (1.26)	2.1 (0.38)	<0.0001
	Best	Static	2.03 (0.92)	1.25 (0.44)	<0.0001
		Dynamic	2.38 (1.13)	1.725 (0.45)	0.001
	Worst	Static	4 (1.58)	2.375 (0.54)	<0.0001
		Dynamic	4.65 (1.42)	2.925 (0.57)	<0.0001
24 hours	Average	Static	2.7 (0.88)	2.1 (0.30)	<0.0001
		Dynamic	3.08 (1.29)	2.25 (0.54)	<0.0001
	Best	Static	2.15 (0.80)	1.77 (0.42)	0.01
		Dynamic	2.55 (1.08)	1.92 (0.35)	<0.0001
	Worst	Static	4.05 (1.68)	2.67 (0.76)	<0.0001
		Dynamic	4.77 (1.64)	3.15 (0.58)	<0.0001

None of the participants in the DUNLuPS group required fentanyl or tramadol as post-operative analgesia (Table 5).

**Table 5:** Analgesic drug consumption in the post-operative period. Values are shown in mean (SD) and compared using Student's t-test. EA: epidural anaesthesia; DUNLuPS: dual ultrasound and nerve stimulator-guided lumbar plexus-sciatic nerve block.

Drugs	EA group	DUNLuPS group	P-value
Paracetamol (mg)	1392.85 (497.34)	1000 (280.97)	0.0026
Diclofenac (mg)	178.84(58.64)	108.871 (42.59)	0.000

## Discussion

In our study, the success of achieving adequate anaesthesia in the DUNLuPS and EA groups when administered by trainees (more than two years of clinical experience) under the supervision of a consultant was 93.47% and 95.65%, respectively, and was comparable. There are numerous approaches to lumbar plexus/sciatic nerve block and the results of our study are limited to the combined US and PNS-guided lumbar plexus block utilizing the Shamrock technique and subgluteal approach for sciatic nerve block. A previous study had shown that the lumbar nerves were identified as hyperechoic structures in more than 50% of cases, making needle visibility, which is also hyperechoic, poor. Neurostimulation improved the identification of the lumbar plexus and enhanced the spread of local anaesthetic [8]. A block failure rate of 9.1% has been described for nerve stimulation-guided lumbar plexus block in a previous study [9].

Failed epidural catheters were replaced in 2.1% and 3.9% of obstetrical patients after combined spinal-epidural and only epidural catheter technique of labour analgesia, respectively, in a retrospective study of 5487 patients [10]. Epidural catheters remain untested in the former and the anaesthesia trainee faces challenges after regression of subarachnoid block (SAB) when a failed epidural necessitates conversion into general anaesthesia. In the usual clinical practice, anaesthetists administer combined spinal epidural anaesthesia and only EA is chosen for patients with low ejection fractions or haemodynamic compromises [11].

The benefit of a shorter sensory and motor block onset time with the DUNLuPS is not of much clinical relevance and is nullified by the delay caused during the performance of the procedure, which was higher in DUNLuPS by around four minutes. The quality of intraoperative anaesthesia in the DUNLuPS group was comparable with that obtained by an intermittent bolus epidural technique. However, the post-operative pain management was superior and the incidence of nausea/vomiting was inferior in the former when compared to the EA group. The failure rate of epidural analgesia has been reported as 30-50% in providing static pain relief in earlier studies [12,13].

Different dosing regimens of epidural analgesia exist for post-operative pain management [14]. In our study, we did not add any adjuvant and administered a bolus dose of 7 ml of 0.2% ropivacaine when the NRS was ≥3. Mehta et al. have reported effective post-operative analgesia with the same regime, but with a higher epidural dose requirement (78.96 ± 6.79 mg in the former vs. 33.60 ± 11.77 mg of ropivacaine in our study) [15].

ED50 (effective dose 50) of 0.5% ropivacaine for lumbar plexus block is 20.4 ml and a similar dose was used by us but the dose and volume of LA to be administered in sciatic nerve block were variable for each patient in our study [7]. Similarly, another study by Diwan et al. showed that the mean volume of local anaesthetic used in ultrasound-guided neurostimulation-aided lumbar plexus block was 19.62 ml [16]. Successful blocks of sciatic nerve have been performed with a volume range of 6.2-20.9 ml based on the concept that dosage varies with different cross-sectional nerve areas (CSA) [17]. The volume of 0.5% ropivacaine administered in the sciatic block in our study was limited by the maximum allowable dose of 3 mg/kg. CSA of sciatic nerve correlates with height and weight of the patients and lower body weight minimizes the maximum allowable volume of local anaesthetic and also necessitates the use of lower volumes.

The limitation of our study is that patient-triggered intermittent administration of 7 ml of 0.2% ropivacaine was the regime of epidural analgesia used by us; research needs to be conducted with different regimes of LA-opioid infusions in epidural catheters to bring out comparisons and similarities of PNBs and central neuraxial blocks (CNBs). The type of anaesthesia administered during previous surgeries was not recorded. Thus, its effect on NRS scores could not be commented on. Previous exposures to anaesthesia could lead to different pain thresholds. The results of this trial could not be generalized to obese or technically challenging patients, as the same group of patients were not included in this study. Incidence of failure rates in interventional procedures is negatively correlated with the experience of an anaesthesiologist. Training and exposure of junior-level trainee anaesthesiologists varies between different centres.

## Conclusions

This study evaluated the competency of 20 anaesthesia trainees in performing DUNLuPS compared to the basic central neuraxial block technique, with 46 patients in each group. After two years of residency and training, the success rates were comparable between the techniques, indicating that third-year residents are competent in DUNLuPS. Implementing a block room for regional anaesthesia can reduce the time trainees spend on nerve blocks. Tailoring anaesthesia training to ensure competency in advanced techniques is essential for improving patient care.
